# Supplementary material for: Utilizing genetic code expansion to modify N-TIMP2 specificity towards MMP-2, MMP-9, and MMP-14
Source: Sci Rep. 2023 Mar 30;13:5186. doi: 10.1038/s41598-023-32019-3 (PMC10063552; doi:10.1038/s41598-023-32019-3)
Supplement: Supplementary file 1 — Supplementary Information. [file 41598_2023_32019_MOESM1_ESM.docx]

**Supplementary Figure 1:** The original image of full length SDS-PAGE of purified N-TIMP2 variants. All proteins were in the expected size of ~15 kDa. Lane 1, N-TIMP2; Lane 2, N-TIMP2-S2DOPA; Lane 3, N-TIMP2-S69DOPA; Lane 4, N-TIMP2-A70DOPA; Lane 5, N-TIMP2-L100DOPA; Lane 6, N-TIMP2-S2HqAlA; Lane 7, N-TIMP2-Y36HqAlA; Lane 8, N-TIMP2-S69HqAlA; Lane 9, N-TIMP2-A70HqAlA. Samples were run on 15% polyacrylamide gel under reducing conditions.
